# The electrophysiological effect of cannabidiol on hERG current and in guinea-pig and rabbit cardiac preparations

**DOI:** 10.1038/s41598-020-73165-2

**Published:** 2020-09-30

**Authors:** Péter Orvos, Bence Pászti, Leila Topal, Péter Gazdag, János Prorok, Alexandra Polyák, Tivadar Kiss, Edit Tóth-Molnár, Boglárka Csupor-Löffler, Ákos Bajtel, András Varró, Judit Hohmann, László Virág, Dezső Csupor

**Affiliations:** 1 Department of Ophthalmology, Faculty of Medicine, Albert Szent-Györgyi Clinical Center, University of Szeged, Szeged, Hungary; 2 Department of Pharmacology and Pharmacotherapy, University of Szeged, Szeged, Hungary; 3 MTA-SZTE Research Group for Cardiovascular Pharmacology, Hungarian Academy of Sciences, Szeged, Hungary; 4 Department of Pharmacognosy, Faculty of Pharmacy, University of Szeged, Eötvös u. 6, Szeged, 672 Hungary; 5 Institute for Translational Medicine, Medical School, University of Pécs, Pecs, Hungary; 6 Department of Pharmacology and Pharmacotherapy, Interdisciplinary Excellence Centre, University of Szeged, Szeged, 6720 Hungary

**Keywords:** Cardiovascular biology, Electrophysiology, Cardiology, Risk factors

## Abstract

Cannabis use is associated with cardiovascular adverse effects ranging from arrhythmias to sudden cardiac death. The exact mechanism of action behind these activities is unknown. The aim of our work was to study the effect of cannabidiol (CBD), tetrahydrocannabinol and 11-nor-9-carboxy-tetrahydrocannabinol on cellular cardiac electrophysiological properties including ECG parameters, action potentials, hERG and I_Kr_ ion channels in HEK cell line and in rabbit and guinea pig cardiac preparations. CBD increased action potential duration in rabbit and guinea pig right ventricular papillary muscle at lower concentrations (1 µM, 2.5 µM and 5 µM) but did not significantly change it at 10 µM. CBD at high concentration (10 µM) decreased inward late sodium and L-type calcium currents as well. CBD inhibited hERG potassium channels with an IC_50_ value of 2.07 µM at room temperature and delayed rectifier potassium current with 6.5 µM at 37 °C, respectively. The frequency corrected QT interval (QT_c_) was significantly lengthened in anaesthetized guinea pig without significantly changing other ECG parameters. Although the IC_50_ value of CBD was higher than literary C_max_ values after CBD smoking and oral intake, our results raise the possibility that hERG and potassium channel inhibition might have a role in the possible proarrhythmic adverse effects of cannabinoids in situations where metabolism of CBD impaired and/or the repolarization reserve is weakened.

## Introduction

Cannabis is the most abused hallucinogenic drug, with an estimated of 150 million consumers worldwide^1^. With the increasingly widespread use of e-cigarettes, the number of people inhaling cannabinoids might even be higher. Moreover, the use of cannabis products for medicinal purposes is increasing globally. The interest for the use of cannabis and cannabis-derived products started following the discovery of the cannabinoid system in the human brain and body and the subsequent reports on new findings on biological activities of cannabinoids on central nervous system and immune functioning. Currently, there are cannabis-based medicines on the market with well-defined medicinal indications, including treatment of nausea and vomiting associated with chemotherapy, anorexia, pain related to cancer, spasticity and pain associated with multiple sclerosis, Dravet and Lennox-Gastaut syndromes. These medicines contain known amounts of CBD and/or THC in pure form or as standardized herbal extract^2^. Besides, the use of CBD-containing products (CBD oil) is very widespread with several, clinically unsupported indications. The intake of cannabinoids, especially CBD, which is enriched in several products, may be higher in case of the consumption of CBD oils than in case of smoking cannabis.

The cardiovascular adverse effects of cannabinoid use have been reported in several case reports, and range from arrhythmias to myocardial infarction and sudden death^3^. According to the results of a cohort study, marijuana smokers have a 4.8-fold increased risk of developing acute myocardial infarction during the first hour of exposure^4^. However, other data do not support the association between cannabis use and cardiovascular events^5^. The most comprehensive study assessed data for 316,397 cannabis users and 20,499,215 non-users, and found that cannabis use is an independent predictor of heart failure^1^. Although the exact mechanisms explaining these observations are unknown, the activities of cannabinoids exerted via the G protein-coupled cannabinoid receptors are supposed to be of key importance. In addition, several studies described the proarrhythmic potency of cannabinoids ranging from ventricular arrhythmias to sudden cardiac death^6–8^. However, the exact association and mechanism of these arrhythmias remain unknown^7^. Besides, certain voltage-gated ion channels like cardiac sodium, calcium^9^ and Kv4.3 channels^10^ might also be related to the reported cardiovascular effects of cannabinoids, but the exact role of these channels has not been studied yet in detail. One of the most important ion channels in cardiac repolarization is the rapid delayed rectifier potassium channel (I_Kr_), which plays a critical role in cardiac repolarization, having a pore-forming subunit encoded by the *hERG* (the human Ether-à-go-go-Related Gene) gene. Inhibitors of I_Kr_ (also called hERG ion channel) are known to lengthen the QT interval, and hence might induce life-threatening arrhythmias. Therefore, formal drug development requires an early screening of whether the potential drug candidates bear any activities on the hERG channels^11^. However, drug effects on cardiac repolarization cannot be accurately estimated by measuring hERG channel currents alone^12^, since drug responses on native I_Kr_ channel and action potential can be different from those measured in hERG.

In the present study cannabidiol (CBD), tetrahydrocannabinol (THC) and 11-nor-9-carboxy-tetrahydrocannabinol (11-nor-9-carboxy-THC), the main metabolite of THC was assessed for their effects on the hERG channels in an in vitro assay. CBD and THC are the major components of cannabis products for medicinal and recreational use, respectively, and since the latter is quickly metabolized to 11-nor-9-carboxy-THC, these three compounds were chosen to be tested in vitro.

Therefore, the aim of our work was to study the in vitro and in vivo effects of CBD a major cannabinoid on cardiac ventricular action potential, on ECG parameters, on the hERG and on other native cardiac transmembrane channels to provide experimental data for the elucidation of their possible adverse cardiac electrophysiological effects.

## Results

**Figure 1:**
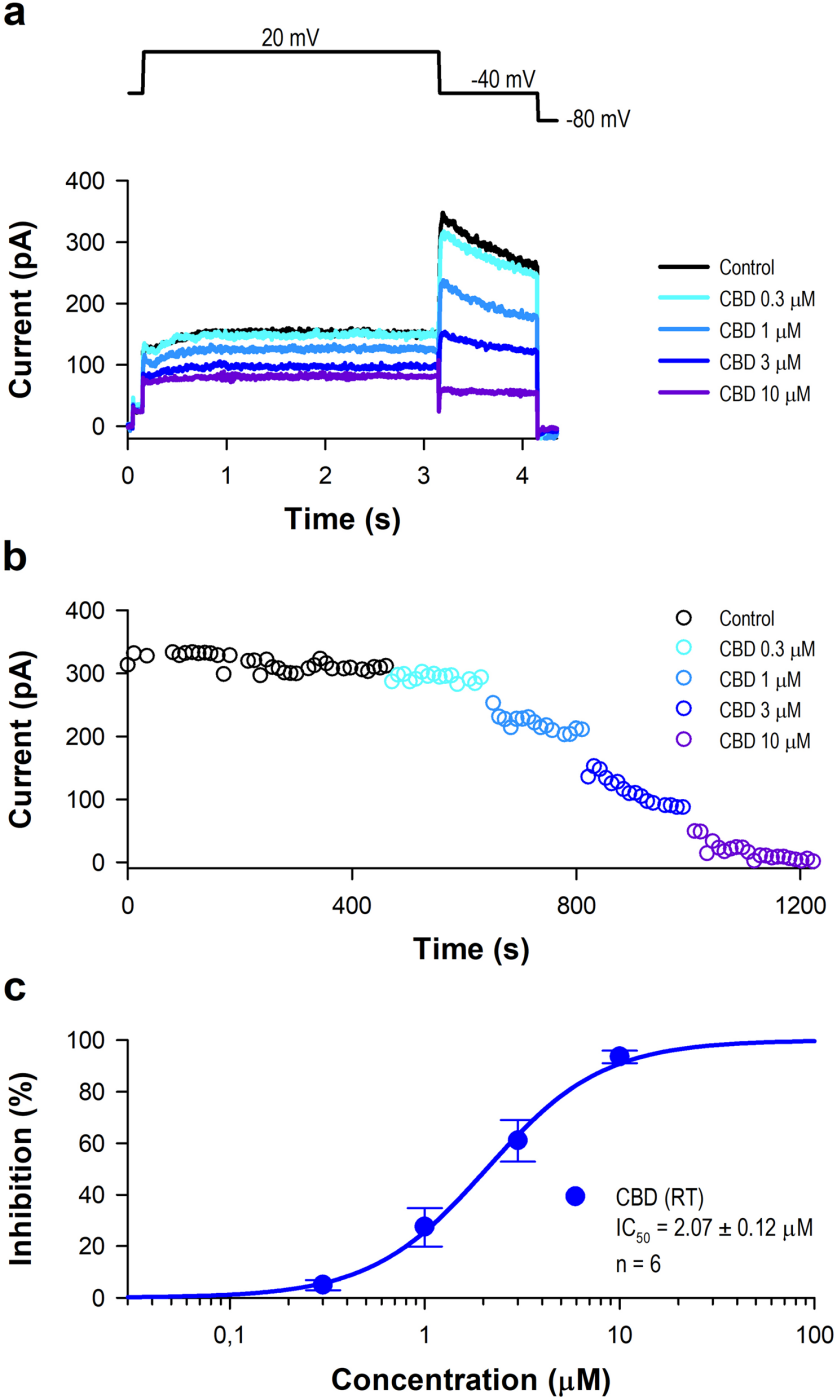
Effect of CBD on hERG current at room temperature. (**a**) Representative current curves obtained from HEK-hERG cells treated with 0.3, 1, 3, and 10 µM CBD. The currents were recorded using the voltage protocol shown at the top of the panel after 3–5 min acute superfusion of the drugs without washout. (**b**) Time-course of the hERG peak tail current amplitude upon the application of different concentrations of CBD. (**c**) Dose–response curves of CBD’s inhibitory activity on the hERG channel.

As our first test shown in Fig. 1, CBD was found to be an inhibitor of the hERG potassium channel with intermediate potency represented by IC_50_ values of 2.07 ± 0.12 µM (n = 6) at room temperature. The IC_50_ values for the inhibition elicited by THC were higher (10.30 ± 0.55 µM, n = 6 at room temperature). 11-Nor-9-carboxy-THC exhibited only a marginal effect (IC_50_ = 65.40 ± 3.82 µM, n = 4 at room temperature).

**Figure 2:**
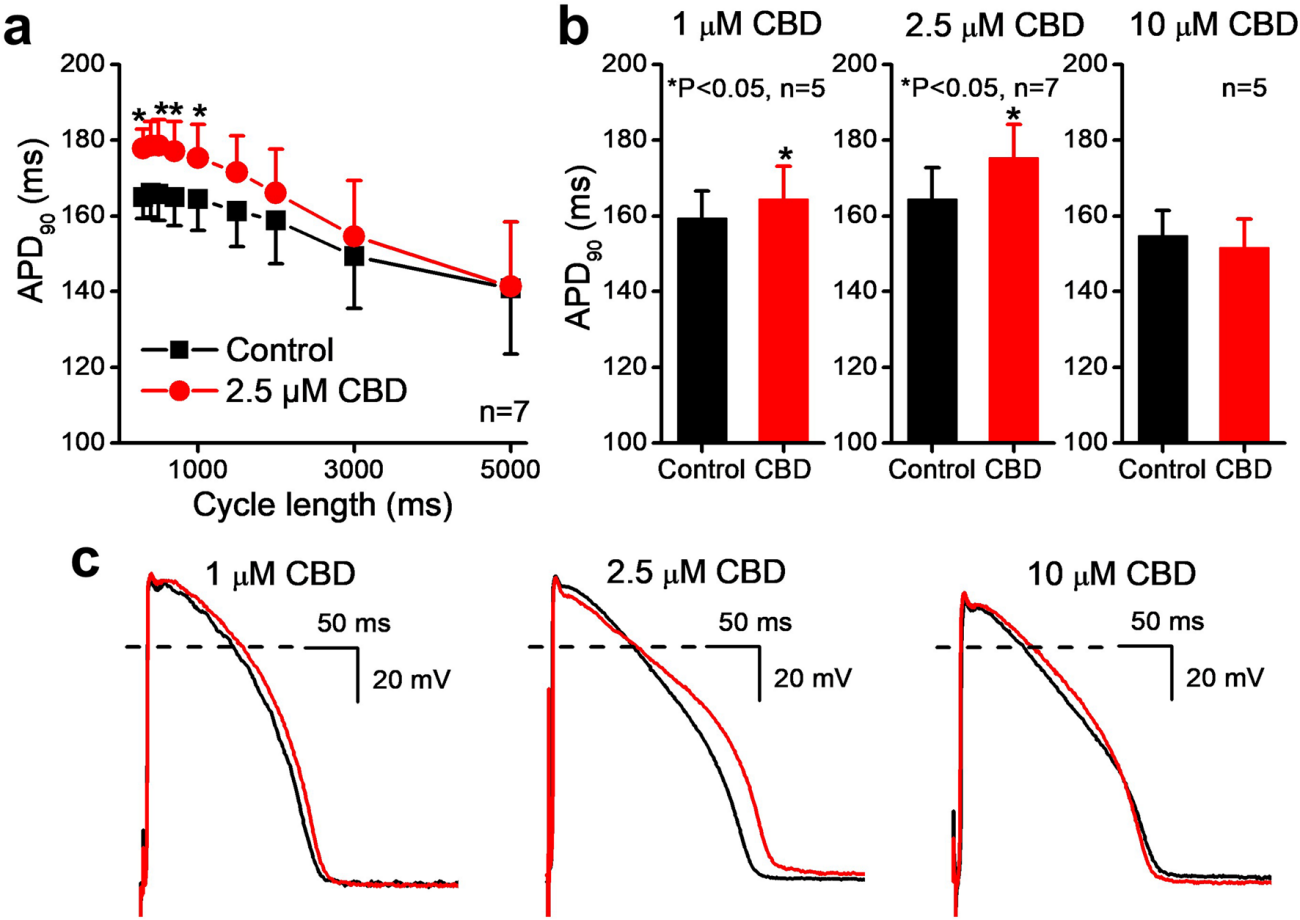
Effect of CBD after 30–50 min acute superfusion of the drug without washout on action potentials recorded from rabbit right ventricular papillary muscle at 37 °C. (**a**) The cycle length-dependent effect of 2.5 µM CBD on the duration of action potentials (APD_90_). Bar diagrams (**b**) indicates the effects of 1 µM, 2.5 µM and 10 µM CBD on the action potential duration during steady-state at 1000 ms cycle length. Original action potential traces are shown on (**c**) recorded at 1000 ms cycle length in control conditions and in the presence of 1 µM, 2.5 µM and 10 µM CBD.

The cardiac cellular electrophysiological effect of the most potent cannabis compound CBD was further studied on various transmembrane ionic currents by the whole-cell configuration of the patch clamp technique in native rabbit ventricular myocytes and on action potentials in rabbit and guinea pig right ventricular papillary muscle by the conventional microelectrode technique and on in vivo ECG studies in anaesthetized guinea pigs. Figure 2 shows that CBD lengthens action potential duration (APD_90_) slightly but significantly at 1 µM and at 2.5 µM. This latter effect depended on the stimulation frequency and vanished at slow pacing rate. At high 10 µM concentration CBD exerted variable effect on repolarization including minimal or no change, shortening and lengthening of APD_90_ resulting statistically not significant alteration of APD. At 1 and 2.5 µM CBD caused triangulation in some experiments but not in others reflected as not significant change in APD_90_–APD_25_. Similar results were obtained in guinea pig papillary muscles where 2.5 and 5 µM CBD increased APD_90_ from 186.2 ± 6.1 ms and 179.9 ± 6.0 ms to 192.2 ± 6.8 and to 191.5 ± 8.9 ms, respectively (p < 0.05, n = 5).

**Figure 3:**
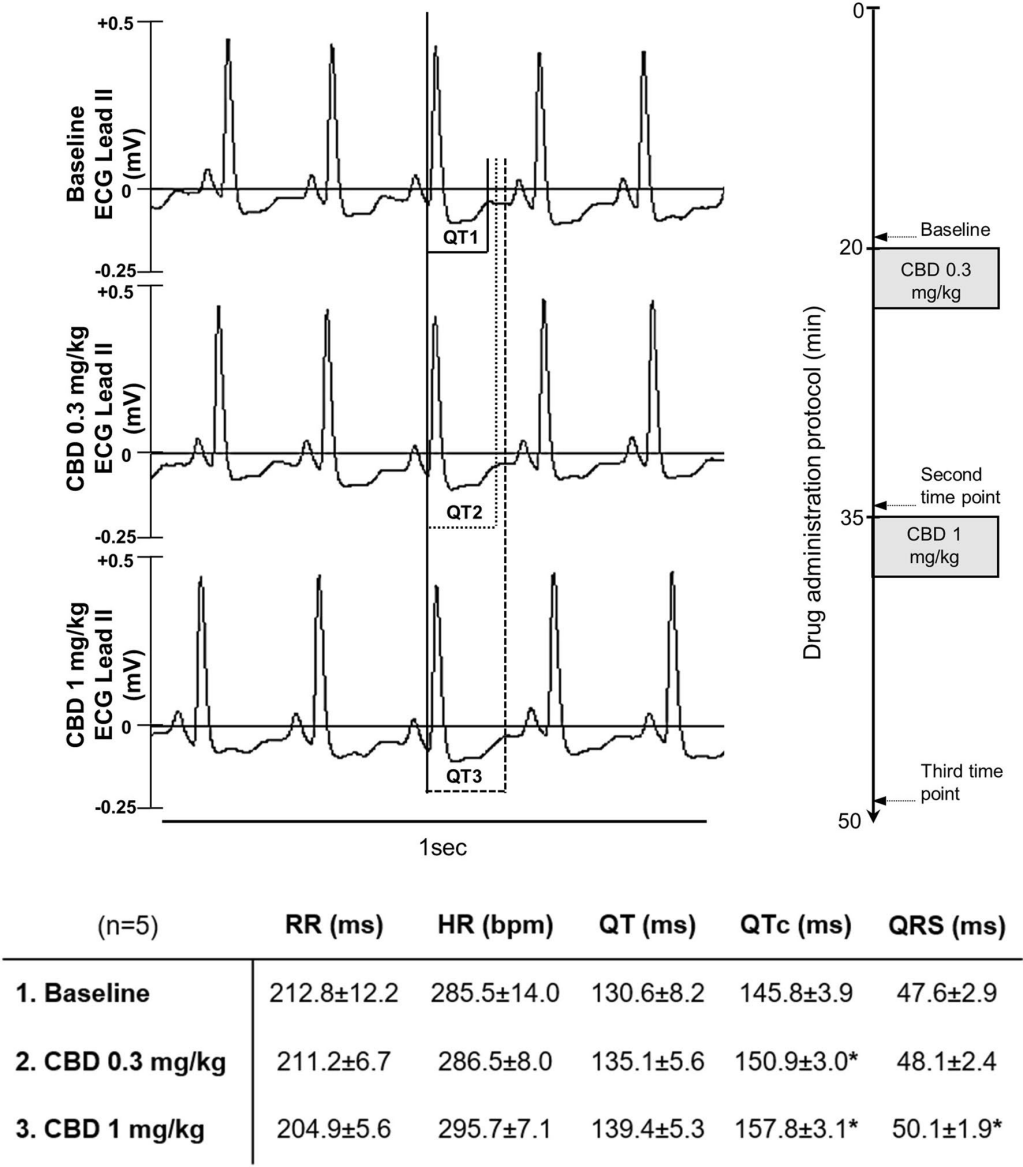
The volume conducted electrocardiogram (ECG lead II) signals in regular sinus rhythm in a pentobarbital anaesthetized (30 mg/kg i.p. bolus injection) guinea pig at three different time points indicated with dashed arrows: **1.** drug-free baseline, value determined from 40 consecutive beats before drug administration; **2.** value determined from 40 consecutive beats 15 min after the 0.3 mg/kg intravenously (iv) administered cannabidiol (CBD) by 2 min bolus; **3.** value determined from 40 consecutive beats 15 min after the 1 mg/kg iv administered CBD by 2 min bolus. RR interval: the time elapsed between two successive R-waves of the QRS signal on the ECG. *HR* heart rate, *QT interval* the time from the start of the Q wave to the end of the T wave, *QTc interval* heart rate corrected QT interval, calculated with a correction method described earlier^27,28^, *QRS interval* the time from the onset to the end of the QRS complex. Table shows the mean ± SE values of the ECG intervals at three different time points. Changes in mean scores over three time points were compared using the repeated measures ANOVA with Bonferroni correction. *p < 0.05 was taken as indicative of a statistically significant difference between values.

In anaesthetized in vivo guinea-pig experiments intravenous administration of 0.3 mg/kg and 1 mg/kg CBD lengthened QTc and QRS intervals in a statistically significant manner without significantly changing other ECG parameter (Fig. 3).

**Figure 4:**
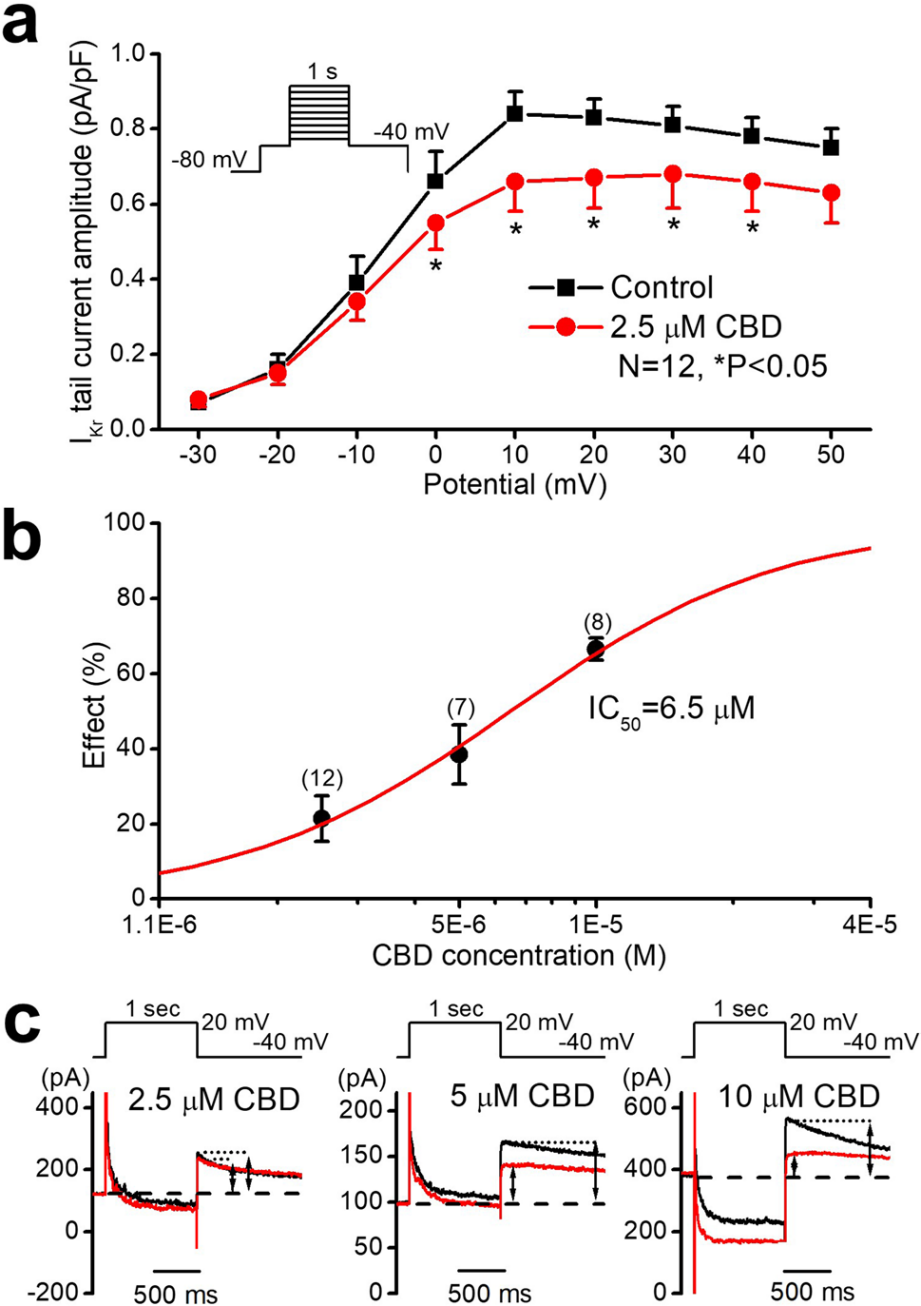
Effect of CBD after 3–5 min acute superfusion of the drug without washout on the rapid delayed rectifier potassium current (I_Kr_) in rabbit left ventricular myocytes at 37 °C. Current–voltage curves show the inhibition of I_Kr_ by 2.5 µM CBD (**a**). (**b**) Displays CBD concentration–response curve indicating an estimated IC_50_ value of 6.5 µM for I_Kr_ blockade. Original I_Kr_ current traces are shown on (**c**) in control conditions and in the presence of 2.5 µM, 5 µM and 10 µM CBD recorded from rabbit left ventricular myocytes after a 1 s long pulse to 20 mV test potential with pulsing cycle length of 20 s. I_Kr_ deactivating tail current was measured at −40 mV. The vertical axis on the left side of the panels shows the absolute current level. The dashed lines refer to the baseline for I_Kr_ tail current level after the test pulse at −40 mV. The arrows indicate the amplitudes of the I_Kr_ tail currents.

Whole-cell patch clamp experiments on rabbit native cardiac ventricular myocytes revealed significant and voltage-dependent inhibition of the rapid delayed rectifier potassium current (I_Kr_) (Figs. 4a,c) with an estimated IC_50_ value of 6.5 µM after a 20 mV 1 s long test pulse and measured at −40 mV as deactivating tail current (Fig. 4b).

**Figure 5:**
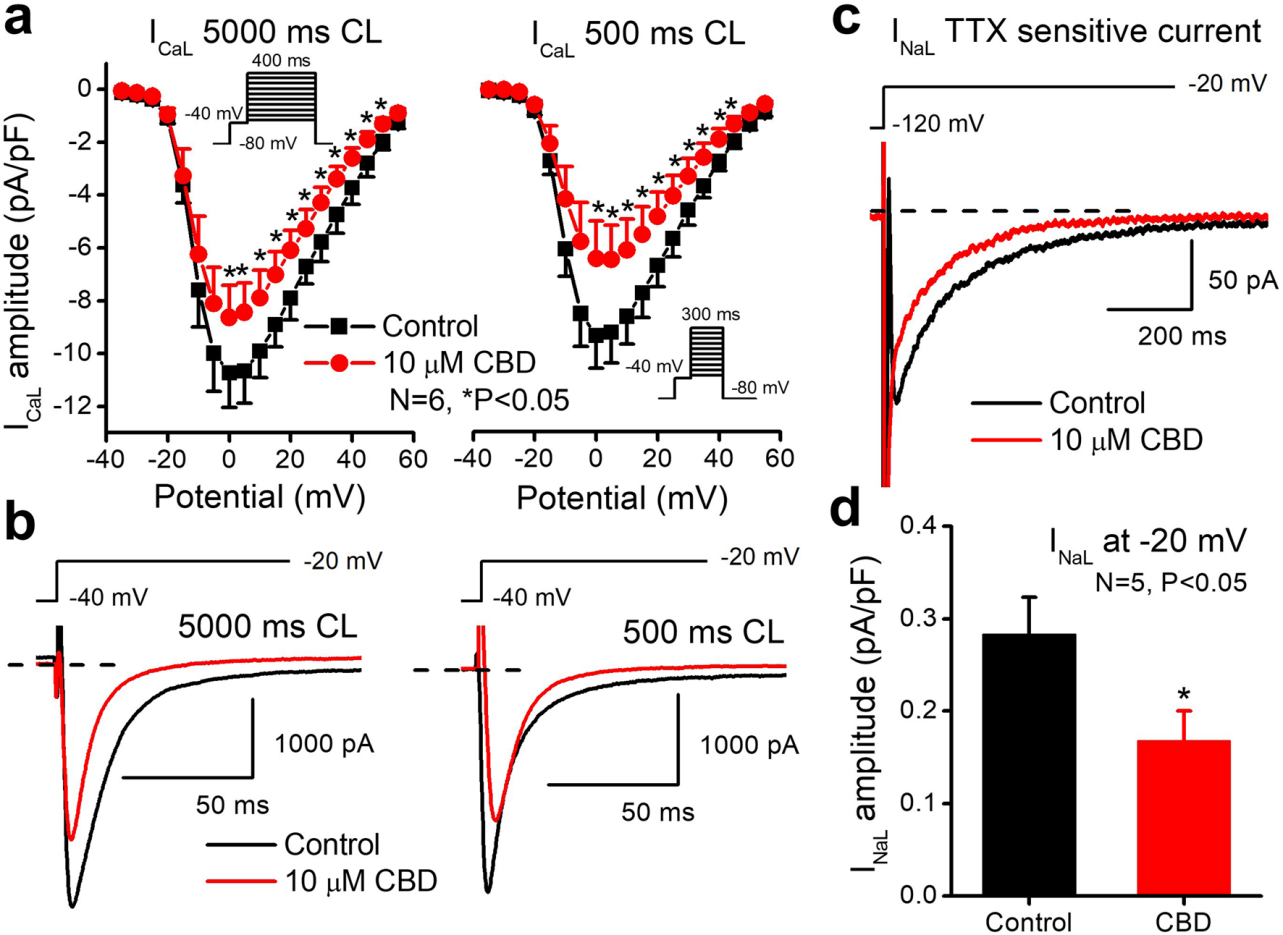
Effect of CBD after 3–5 min acute superfusion of the drug without washout on L-type calcium (I_CaL_) and on the late sodium (I_NaL_) currents in rabbit left ventricular myocytes at 37 °C. On (**a**) current–voltage curves show the inhibition of I_CaL_ by 10 µM CBD at 5000 ms (left) and at 500 ms (right) cycle lengths. Original I_CaL_ current traces are shown on (**b**) in control conditions and in the presence of 10 µM CBD recorded from rabbit left ventricular myocytes at 5000 ms (left) and at 500 ms (right) cycle lengths at 0 mV test potential. TTX sensitive current (I_NaL_) traces (**c**) and a bar diagram (**d**) show the inhibition of I_NaL_ by 10 µM CBD measured at −20 mV in rabbit left ventricular myocytes.

The observation that high (10 µM) concentration of CBD did not further lengthened APD prompted us to study the possible effect of CBD on inward L-type Ca^2+^ (I_CaL_) and late inward Na^+^ (I_NaL_) currents. As Fig. 5a,b show, 10 µM CBD decreased I_CaL_ significantly and in a frequency-dependent manner. In addition, 10 µM CBD also significantly inhibited I_NaL_ by 41.5% at −20 mV (Fig. 5c,d).

## Discussion

The main result of our study is that CBD lengthens repolarization at low and does not change it statistically significant manner at higher concentrations. This effect on repolarization in rabbit papillary muscle can be best explained by the multiple ion channel effects of CBD. Accordingly, at lower concentrations (1, 2.5 and 5 µM) I_Kr_ depression results in lengthening of APD_90_, which is counterbalanced by inward Ca^2+^ and Na^+^ currents inhibition at 10 µM. Similar effect was earlier described by quinidine, an antiarrhythmic drug, with reported proarrhythmic property^13,14^. The I_Kr_ inhibition by CBD, which is consistent with the hERG blockade, is most probably a direct effect on the channel but the effect of CBD on I_NaL_ and on I_CaL_ can be either direct or receptor mediated, as well. It needs more research to be established.

The higher IC_50_ values of THC and 11-nor-9-carboxy-THC than that of CBD in hERG current measurement in our study does not necessarily mean that CBD is more potent in other type of experiments since potency can differ from a target to another^12^. However, in our study due to technical limitations we focused our investigations to examine the effects of CBD in more depth.

Some previous study with cannabinoids showed effects on various transmembrane ion channels such as inward sodium^9,15^, inward calcium^9^, outward transient current^16^ and human Kv1.5 and Kv4.3 channels^10,17^. Our results in rabbit ventricular myocytes are in good agreement with those reported by Al Kury et al. on inward calcium and sodium currents in rat ventricular myocytes^9^. In a previous study hERG channel inhibition and QT lengthening were also reported in anaesthetized rats^18^ by a synthetic cannabinoid compound (JWH-030). This synthetic cannabinoid compound differs from those investigated by us and inhibited hERG channel with a relatively high IC_50_ (88.36 µM). In addition, in rat ventricle hERG/I_Kr_ seems not so important to control repolarization than Kv4.2 and Kv1.5 channels. Therefore, the cannabinoid-evoked QT changes in rat most likely can be attributed to Kv1.5 and Kv4.2 rather than hERG channel inhibition. It is worth to note that in the same study^18^ a cannabinoid derivate JWH-030 did not change APD in low but shortened it at high (30 µM) concentration. Therefore, the results of our study is in partial agreement with these earlier reports and the differences are best explained by different preparations, chemical differences of the studied compounds and experimental conditions.

The IC_50_ values reported here together with the C_max_ values of CBD allow the assessment of cardiovascular risks of this compound. Based on the comparison of hERG or I_Kr_ activity, cardiac action potential duration, and QT prolongation against QT effects and reports of arrhythmogenic (torsade de pointes) potential of 100 drugs, a margin of at least 30-fold between hERG IC_50_ and C_max_ was proposed to an acceptable degree of safety from arrhythmogenesis^19^. According to human pharmacokinetic data, the C_max_ values for CBD might reach 0.35 µM and 0.58 µM after CBD smoking (19.2 mg) and oral intake (400 mg), respectively^20^. In our experiments CBD had an inhibitory effect on both the hERG channel and I_Kr_ activity, with an IC_50_ value higher than literary C_max_ values in patients. Considering the IC_50_ values for I_kr_ and hERG channel inhibition in our experiments (6.5 and 2.07 µM, respectively), the ratios of IC_50_ and C_max_ values are in the range of 3.57–18.57. This safety margin (below 30) suggests a potential proarrhythmic risk in human setting. However, previous clinical reports documented no significant QT and QT_c_ prolongation in patients after CBD administration^21^. Also, in another study it was found that long term Sativex (CBD + THC) treatment evoked T wave changes only 1 out of 146 patients^22^. This might be explained by the effects of CBD on other ion channels than hERG and I_Kr._(eg. I_CaL_ and I_NaL_). However, in patients who have slower drug elimination due to certain diseases or in case of concurrent use of medicines inhibiting the metabolism of CBD, higher C_max_ values may develop and the risk of arrhythmia might be increased^23^. Moreover, when co-administered with pharmacological agents affecting cardiac repolarization, as well as in certain pathophysiological conditions such as hypokalaemia, or diseases like LQT syndrome, HCM, diabetes mellitus or heart failure where cardiac repolarization reserve or drug metabolism is impaired, CBD may have an additive effect, further increasing the proarrhythmic risk and the possibility of sudden cardiac death. Such additive effect was reported in both in experiments^24^ and in patients^25^. The cardiovascular effects of CBD may only partly be interpreted on its effects on hERG and I_Kr_ ion channels, the cardiovascular safety of this compound may be influenced by its activities on other ion channels. Further studies are needed to assess the effects of other cannabinoids as well, and the in vivo relevance of these results, with special focus on the benefit-risk assessment of products with different cannabinoid content.

## Methods

The hERG channel current was measured by planar technology in HEK 293 cell line by the whole-cell configuration using an automated patch clamp system (Patchliner, Nanion Technologies GmbH., Munich, Germany) at room temperature as described previously^12^. The following solutions were used during automated patch-clamp recording (compositions in mM): internal solution: KCl 50, NaCl 10, KF 60, EGTA 20, HEPES 10, pH 7.2 (KOH); external solution: NaCl 140, KCl 4, glucose-monohydrate 5, MgCl_2_ 1, CaCl_2_ 3, HEPES 10, pH 7.4 (NaOH). The voltage protocol for hERG ion channel started with a short (100 ms) −40 mV step to establish the baseline region. A depolarizing step was applied to the test potential of 20 mV for 3 s, and then the cell was repolarized to −40 mV (1 s) to evoke outward tail current. The peak tail current was corrected the leak current defined during the first period to −40 mV. Holding potential was −80 mV. The pulse frequency was approximately 0.1 Hz. Recording started in external solution. After this control period, increasing concentrations of the test compound were applied, in order to record a complete concentration–response curve.

The action potential measurements were carried out in rabbit and guinea pig right ventricular papillary muscles by the conventional microelectrode techniques at 37 °C as described in detail earlier^12,26^. Isolated muscle preparations obtained from the right ventricle were individually mounted in a tissue chamber while superfused with oxygenated modified Locke’s solution containing (in mM): NaCl 128.3, KCl 4, CaCl_2_ 1.8, MgCl_2_ 0.42, NaHCO_3_ 21.4 and glucose 10 (pH 7.35–7.4) and stimulated through a pair of platinum electrodes with constant cycle length of 1000 ms. In case of cycle length-dependent measurements stimulation with different constant cycle lengths ranging from 300 to 5000 ms were also applied. Transmembrane potentials were recorded using conventional glass microelectrodes, filled with 3 M KCl and having tip resistances of 5–20 MΩ, connected to the input of a high impedance electrometer (Experimetria, type 309, Budapest, Hungary). The analog action potential signals were digitized with analogue-to-digital converters (ADA 3300, Real Time Devices Inc., State College, PA, USA) under software control (APES home-made software).

Transmembrane ion currents in native rabbit ventricular myocytes were measured by the whole-cell configuration of the patch clamp technique at 37 °C (Axopatch 200B, Molecular Devices Inc., Sunnyvale, CA, USA) as described in detail earlier^12^. Rapid delayed rectifier potassium current (I_Kr_), was recorded in HEPES-buffered Tyrode’s solution containing (in mM) NaCl 144, NaH_2_PO_4_ 0.33, KCl 4.0, CaCl_2_ 1.8, MgCl_2_ 0.53, glucose 5.5 and HEPES 5.0, at pH of 7.4. The composition of the pipette solution (in mM) was the following: KOH 110, KCl 40, K_2_ATP 5, MgCl_2_ 5, EGTA 5, and HEPES 10 (pH was adjusted to 7.2 by aspartic acid). 1 µM nisoldipine and 0.5 µM HMR-1556 (the selective blocker of the slow delayed rectifier K^+^ current—I_Ks_) were added to the external solution to eliminate I_CaL_ and I_Ks_, respectively. I_Kr_ was determined as tail current at −40 mV after the end of 1 s long depolarizing pulses ranging from −30 to +50 mV with pulsing cycle length of 20 s. The L-type calcium current (I_CaL_) was recorded in HEPES-buffered Tyrode’s solution supplemented with 3 mM 4-aminopyridine. A special solution was used to fill the micropipettes (composition in mM: CsCl 125, TEACl 20, MgATP 5, EGTA 10, HEPES 10, pH was adjusted to 7.2 by CsOH). I_CaL_ current was evoked by 400 ms long depolarizing voltage pulses to various test potentials ranging from −35 to +55 mV with pulsing cycle length of 5 s. The holding potential was −80 mV. A short prepulse to −40 mV served to inactivate Na^+^ current. The sodium current was activated by 2 s long depolarizing voltage pulses to −20 mV from the holding potential of −120 mV with pulsing cycle length of 5 s. After 5–7 min incubation with CBD the external solution was replaced by that containing 20 µM TTX. TTX at this concentration completely blocks the late sodium current (I_NaL_). The external solution was HEPES-buffered Tyrode’s solution supplemented with 1 µM nisoldipine, 0.5 µM HMR-1556 and 0.1 µM dofetilide in order to block I_CaL_, I_Ks_ and I_Kr_ currents. The composition of the pipette solution (in mM) was: KOH 110, KCl 40, K_2_ATP 5, MgCl_2_ 5, EGTA 5, HEPES 10 (pH was adjusted to 7.2 by aspartic acid).

ECG recordings were taken from adult guinea-pigs of both sexes (600–800 g) anaesthetized by intraperitoneal 30 mg/kg pentobarbital and I–III leads were recorded after 15 min of cumulative intravenous administration of CBD into the jugular vein^27^.

### Statistics

All data are expressed as means ± SEM. Statistical analysis was performed with Student’s *t *test for paired data. The results were considered statistically significant when p was < 0.05.

### Animal ethics statement

All experiments performed in rabbit and guinea pig ventricular preparations were carried out in compliance with the Guide for the Care and Use of Laboratory Animals (USA NIH publication NO 85-23, revised 1996) and conformed to the Directive 2010/63/EU of the European Parliament. The protocols have been approved by the Ethical Committee for the Protection of Animals in Research of the University of Szeged, Szeged, Hungary (approval number: I-74-24-2017) and by the Department of Animal Health and Food Control of the Ministry of Agriculture and Rural Development (authority approval number XIII/3331/2017).
